# Efficacy of MAKO robot-assisted total knee arthroplasty compared with conventional total knee arthroplasty in patients with knee joint diseases: a systematic review and meta-analysis

**DOI:** 10.3389/fbioe.2026.1892225

**Published:** 2026-08-18

**Authors:** Tao Zhang, Chao Lou, Dan Wu, Jinhe Ying, Jianlong Chen

**Affiliations:** 1 Department of Orthopedic Surgery, Lishui Municipal Central Hospital, Lishui, Zhejiang, China; 2 Department of Medical Examination Center, Qingyuan County People’s Hospital Medical Examination Center, Qingyuan, Zhejiang, China

**Keywords:** MAKO robot, meta-analysis, robot-assisted, systematic review, total knee arthroplasty

## Abstract

**Objective:**

To systematically evaluate the efficacy of MAKO robotic-arm assisted total knee arthroplasty (MAKO-TKA) in patients with knee diseases.

**Methods:**

We searched the CNKI, Wanfang, Vip, China Biomedical Literature Database, PubMed, Cochrane Library, Embase, and Web of science database from inception to July 2026. We included comparative studies (non-randomized controlled studies) involving patients with knee disorders undergoing MAKO robot-assisted TKA (the experimental group received MAKO robot-assisted TKA, and the control group received conventional TKA). Data were analyzed using RevMan 5.2 software.

**Results:**

A total of nine studies involving 937 patients were included, comprising seven retrospective case-control studies and two before-and-after self-comparison studies. Meta-analysis showed that, compared with conventional TKA, the MAKO robot-assisted TKA group had a significantly smaller hip-knee-ankle (HKA) angle (MD = −0.96, 95% CI = −1.26 to −0.65, P < 0.0001) and significantly higher knee function scores (SMD = 0.46, 95% CI = 0.01 to 0.91, P = 0.04). Although pain scores tended to be lower in the MAKO group, the difference between the two groups was not statistically significant (SMD = −0.74, 95% CI = −1.55 to 0.06, P = 0.07). No statistically significant differences were observed in range of motion (ROM) (MD = 0.86, 95% CI = −0.23 to 1.94, P = 0.12) or Forgotten Joint Score (FJS) (SMD = 1.06, 95% CI = −0.15 to 2.27, P = 0.09). Subgroup analysis by follow-up duration revealed that, in the ≤6 months subgroup, MAKO robot-assisted TKA was associated with higher knee function scores (SMD = 6.40, 95% CI 0.48 to 12.32, P = 0.03); however, the test for subgroup differences did not reach statistical significance, indicating that follow-up duration could not be considered an explanatory factor for the heterogeneity observed in knee function scores.

**Conclusion:**

MAKO robot-assisted TKA showed statistically significant, standardized improvements in the HKA angle, and functional knee scores compared to conventional TKA. No conclusive advantage was found for ROM, pain or FJS. These findings support the prioritized use of this technology in cases demanding high precision, such as complex deformities, and highlight the need for standardized outcome measures in future prospective studies.

## Introduction

The use of robot-assisted total knee arthroplasty (RA-TKA) is increasing with advances in total knee arthroplasty. Robots improve surgical accuracy by creating more accurate and precise total knee arthroplasty (TKA) by taking into account patient-specific anatomy (Tang et al., 2024), and have been shown to improve functional outcomes and reduce pin-track related complications (Baek et al., 2024a; Woelfle et al., 2024). The MAKO robotic-arm assisted system (Stryker, USA) is a widely recognized and clinically adopted robotic platform for joint replacement, and represents one of the most frequently utilized orthopedic surgical robots across several countries. In recent years, with the advancement of robot-assisted technology, precision targeting based on individual patient anatomy and the concept of functional alignment have been progressively incorporated into preoperative planning and intraoperative execution for TKA. Recent studies have demonstrated that robot-assisted TKA can enhance the accuracy of prosthetic component positioning and restoration of lower-limb mechanical alignment, while also facilitating the implementation of personalized alignment strategies. However, whether these technical improvements can be consistently translated into sustained long-term clinical benefits remains a matter of debate (Giovanoulis et al., 2025; Seracchioli et al., 2026; Kafelov et al., 2023). Relevant studies have shown that MAKO robot-assisted TKA was effective in reducing patients’ postoperative pain compared with conventional TKA, and patients who underwent MAKO robot-assisted TKA had better overall health and increased vitality at 2 years postoperatively (Baek et al., 2024b). A study by Kim et al. found that the increased cost associated with robot-assisted TKA did not lead to an improvement in outcomes, and recommended against its widespread use (KIM et al., 2020). The aforementioned studies primarily focus on the overall efficacy of robot-assisted techniques, encompassing various robotic platforms and different alignment strategies. The findings suggest that robot-assisted techniques confer advantages in terms of radiological accuracy; however, no consensus has been reached regarding clinical outcomes, including pain relief, functional scores, range of motion, and patient satisfaction (Maman et al., 2024).

With the annual increase in TKA procedures, greater clinical emphasis is being placed on the reproducibility of alignment and prosthesis positioning, perioperative tissue protection, and consistent benefits in patient-reported outcomes. Previous studies suggest that postoperative deviations in alignment and prosthesis position may be associated with adverse outcomes, making the improvement of osteotomy and implantation accuracy and the reduction of outliers a key driver for the development of computer navigation and robot-assisted surgery (HADI et al., 2015). New technologies represented by robot-assisted TKA typically employ preoperative 3D modeling and intraoperative real-time feedback to confine the osteotomy within planned boundaries, thereby demonstrating advantages in radiographic alignment and prosthesis implantation accuracy. Systematic reviews and meta-analyses indicate that robot-assisted TKA generally outperforms conventional manual TKA in metrics such as limb alignment and implantation precision; however, inconsistencies may still exist in functional outcomes across different follow-up stages and assessment scales (Fu et al., 2024). Taking MAKO as an example, some studies have reported its potential advantages in early clinical outcomes and the achievement of alignment planning (Ma et al., 2024). Other research has observed possible benefits of robot-assisted TKA in early pain control and accelerated recovery/shortened hospital stay, although whether this advantage can be consistently translated into medium-to long-term functional improvement and satisfaction requires further validation from higher-quality studies (Kayani et al., 2018). Robot-assisted technology also has objective drawbacks, including higher equipment acquisition and per-case consumable costs, potential demands on operating room space, maintenance, and engineering support, and a learning curve that may prolong surgical time during the initial adoption phase (Dretakis and Koutserimpas, 2024). For CT-dependent systems, although some studies suggest the radiation dose is generally within an acceptable range, the additional examination cost and radiation exposure from preoperative CT must still be considered (Saad et al., 2024). Previous research has also summarized specific risks related to robotics, such as pin-related complications. Although the overall incidence is low, these should be included in patient informed consent and risk management during widespread adoption (Thomas et al., 2022).

Therefore, focusing specifically on the MAKO platform, this study aims to systematically review and perform a meta-analysis on the efficacy differences between MAKO robot-assisted TKA and conventional TKA in patients with knee joint diseases. Specifically, it compares postoperative outcomes including the Hip-Knee-Ankle Angle (HKA angle), pain levels, knee scores, Range of Motion (ROM), and Forgotten Joint Scores (FJS). Furthermore, subgroup analyses based on follow-up time will be conducted to explore time-dependent differences in knee scores. MAKO robot-assisted technology may improve postoperative coronal alignment recovery by enhancing intraoperative positioning and prosthesis implantation accuracy, and may further influence patients’ functional recovery and quality of life. However, whether these radiological advantages translate into consistent clinical benefits remains controversial. This study comprehensively analyzed the HKA angle, pain, knee function scores, ROM, and FJS to evaluate the clinical value of MAKO robot-assisted TKA and to provide evidence-based support for the rational application of robot-assisted technology.

## Methods

### Inclusion and exclusion criteria

Inclusion criteria: The study types were limited to retrospective case-control studies and self-controlled before-and-after studies, and the language was restricted to Chinese and English. Given the limited number of high-quality RCTs, and to avoid evidence insufficiency or omission of the best currently available evidence by restricting to RCTs only, this study prospectively allowed the inclusion of non-randomized studies in its criteria. It is acknowledged that the level of evidence for causal inference in such study designs is generally lower than that of RCTs, and they may be subject to selection bias and confounding factors; The study population comprised patients with knee joint diseases (including knee osteoarthritis, valgus deformity, and varus deformity) who underwent primary TKA, without restrictions on age, sex, or race. The intervention was MAKO robot-assisted TKA for the experimental group and conventional TKA for the control group. The outcome measures were required to include at least one of the following: (1) HKA angle; (2) pain, assessed using the visual analog scale (VAS); (3) knee function scores; (4) ROM; and (5) FJS. Exclusion criteria were: incomplete data; duplicate publications or studies for which the full text could not be obtained; and review articles, conference abstracts, case reports, or animal studies.

### Search strategy

Computerized searches were performed on China National Knowledge Infrastructure (CNKI), Wanfang, Vip, China Biomedical Literature Database, PubMed, Cochrane Library, Embase, and Web of science database, all from inception to July 2026.

The search terms are: “Arthroplasty, Replacement, Knee” OR “total knee arthroplasty” OR “total knee replacement”; “MAKO robotic-assisted” OR “MAKO”. A combination of subject terms and free words was used to simultaneously search for references incorporated into the literature. The English search strategy is exemplified by pubmed: (((Arthroplasty, Replacement, Knee [MeSH Terms]) OR (total knee arthroplasty [Title/Abstract])) OR (total knee replacement[Title/Abstract])) AND ((MAKO robotic-assisted[Title/Abstract]) OR (robotic-assisted[Title/Abstract])). The detailed search strategy and results are presented in Supplementary Table S1.

### Literature screening and data extraction

Literature screening was first carried out automatically by the NoteExpress computerized literature management system, and the duplicate literature was preliminarily screened by reading the title, abstract, etc. After the initial screening, the full text was further read to exclude the literature that did not meet the inclusion criteria. Data extraction was performed back-to-back by two researchers according to the pre-defined inclusion and exclusion criteria, and any disagreement was resolved by discussion or with the assistance of a third researcher. Data extraction included title, author, year of publication, study type, disease type, age, sample size, follow-up time, and outcome indicators.

### Literature quality assessment

The methodological quality of the included studies was independently appraised by two researchers using the Joanna Briggs Institute (JBI) Critical Appraisal Checklist for Quasi-Experimental Studies (Joanna Briggs Institute, 2020). This tool comprises nine fixed criteria assessing the clarity of the research question, presence of a control group, comparability of groups, identification of confounders, description of interventions, use of blinding, reporting of follow-up, reliability of outcome measurements, and appropriateness of statistical analysis. Each item was judged as “Yes”, “No”, “Unclear”, or “Not Applicable”. Any disagreements were resolved through discussion or by consultation with a third researcher.

### Statistical methods

RevMan5.2 statistical software was used to analyze the clinical heterogeneity by adopting I^2^ test. When P ≥ 0.05 and I^2^<50%, it indicated that there was no statistical heterogeneity among the studies, and the fixed-effect model was used for Meta-analysis; conversely, it indicated that there was statistical heterogeneity among the studies, and the source of difference was first analyzed, and if there was no obvious clinical difference and no definite source of statistical difference could be found, the random-effects model was used for Meta-analysis. HKA angle, pain, knee score, ROM, and FJS score indexes included in this study were continuous variables, so standardized mean difference (SMD) and its 95% confidence interval (CI) were used as effect sizes. P < 0.05 was considered a statistically significant difference. A leave-one-out sensitivity analysis was performed using the ‘meta’ package of STATA 18 MP statistical software, whereby each included study was sequentially excluded and the meta-analysis was re-calculated, to evaluate the influence of individual studies on the pooled effect size and the robustness of the overall findings. The results were considered to demonstrate satisfactory robustness if the direction and statistical significance of the pooled effect size did not exhibit substantial alteration following the sequential exclusion of each study.

## Results

### Baseline characteristics of included studies and quality assessment results

**FIGURE 1:**
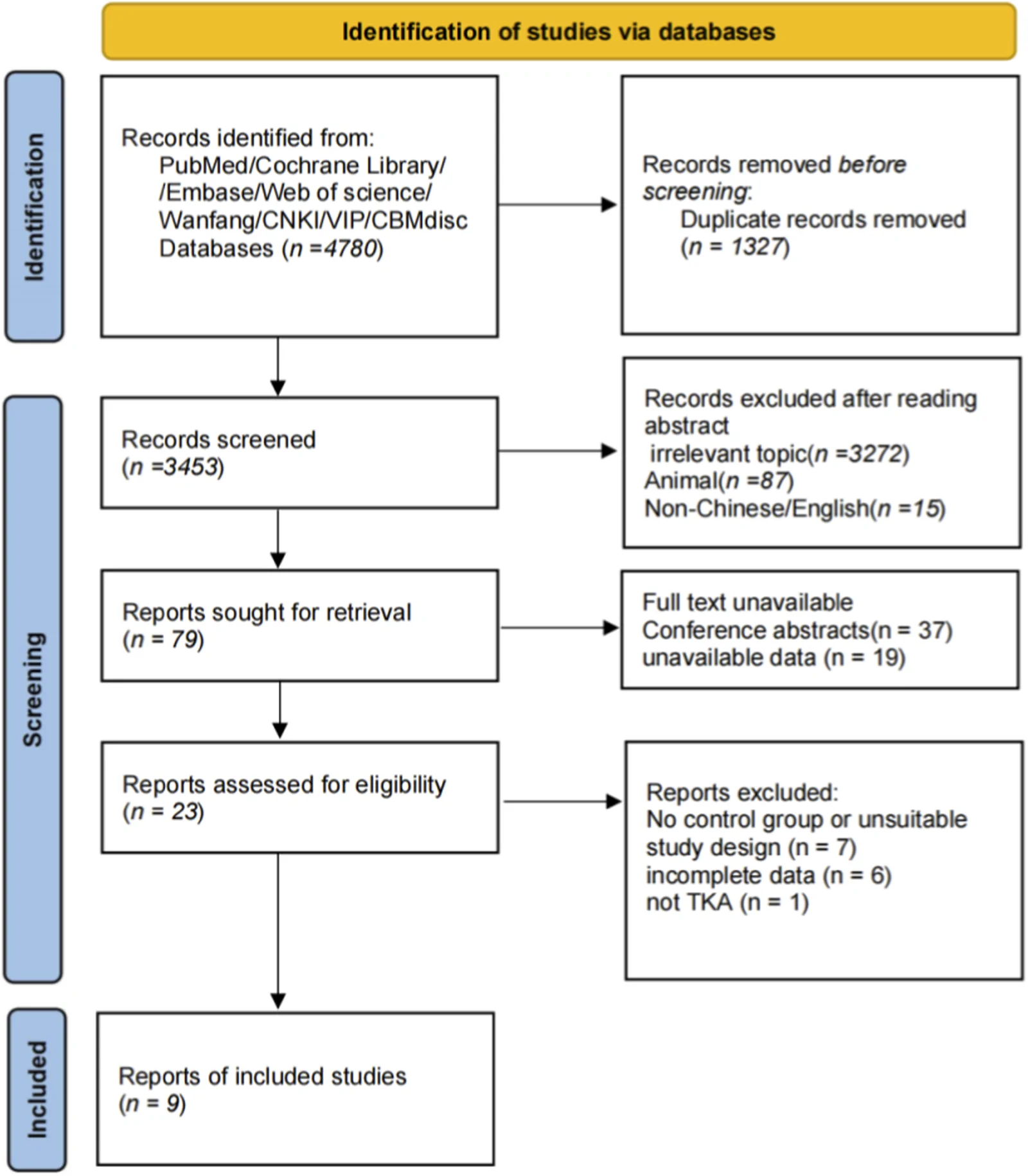
Flowchart of the literature screening process.

A systematic search of PubMed, Cochrane Library, Embase, Web of Science, Scopus, CNKI, Wanfang, and VIP databases yielded 4,780 potentially relevant records. Following the removal of 1,327 duplicate records, 3,453 records remained for initial screening (title and abstract review). During this phase, 3,374 records were excluded due to irrelevance to the topic (n = 3,272), animal studies (n = 87), and non-Chinese/English publications (n = 15), leaving 79 records for full-text retrieval. Of these, 56 records were unobtainable in full text, including conference abstracts without subsequent full-text publication (n = 37) and studies with unavailable data (n = 19). Consequently, 23 full-text articles were assessed for eligibility during the secondary screening. Following this, 14 articles were excluded due to absence of a control group or non-conforming study design (n = 7), incomplete data (n = 6), and interventions other than total knee arthroplasty (n = 1). Ultimately, 9 studies (Ma et al., 2024; Liu et al., 2024; Zhang et al., 2023; Liu et al., 2023; Ru and He, 2023; Zhang et al., 2022; Mu et al., 2022; Xu et al., 2022; Nam et al., 2022) meeting the inclusion criteria were included in the systematic review. The literature screening process is illustrated in Figure 1.

### Results of the baseline characteristics and methodological quality assessment of the included studies

A total of 9 studies (Ma et al., 2024; Liu et al., 2024; Zhang et al., 2023; Liu et al., 2023; Ru and He, 2023; Zhang et al.; Mu et al., 2022; Xu et al., 2022; Nam et al., 2022) were included, comprising 3 English-language articles and 6 Chinese-language articles. Among these, there were 7 retrospective case-control studies and 2 self-controlled before-and-after studies. A total of 937 patients with knee joint diseases undergoing primary total knee arthroplasty were involved, including 473 patients in the experimental group and 464 patients in the control group. The basic characteristics of the literature are shown in Table 1. Methodological quality was assessed using the Joanna Briggs Institute (JBI) appraisal tools for quasi-experimental studies, with the results detailed in Table 2. Overall, the included studies demonstrated adequate reporting in terms of definition of the research question, description of interventions, reporting of follow-up data, measurement of outcome indicators, and statistical analysis methods (meeting criteria 1, 5, 7, 8, and 9). In the 7 case-control studies, a control group was established in all studies and baseline comparability was described (meeting criteria 2 and 3). However, only a subset of studies clearly identified and described potential confounding factors (criterion 4), and none of the studies implemented blinding of participants, intervention providers, or outcome assessors (criterion 6). Therefore, although the included studies exhibited good completeness of reporting in certain methodological aspects, there remains a potential risk of bias, particularly in terms of confounding control and blinding implementation.

**TABLE 1:** Basic characteristics of the included literature.

Author and year	Type of study	Disease type	Sample size		Age		Follow-up time	Outcome indicators and measurement tools
			MA-TKA	CM-TKA	MA-TKA	CM-TKA		
Liu et al. (2024)	Retrospective case-control	Osteoarthritis of the knee	45	47	66.4 ± 5.8	68.7 ± 6.7	6 months	HKA angle; Pain (VAS); ROM; FJS scores
Zhang et al. (2023)	Retrospective case-control	Osteoarthritis of the knee	41	37	66.80 ± 7.48	67.46 ± 6.10	3 months	HKA angle
Liu et al. (2023)	Retrospective case-control	Osteoarthritis of the knee	75	153	65.8 ± 6.8	66.9 ± 7.4	12 months	Pain (VAS); Knee scores (AKS); ROM; FJS scores
Ru and He (2023)	Before and after self-comparison	Osteoarthritis of the knee	73	-	67.8 ± 6.6	-	6 months	Pain (WOMAC); Knee scores (WOMAC)
Zhang et al. (2022)	Retrospective case-control	knee valgus	16	16	59.3 ± 8.3	60.6 ± 8.4	6 months	Pain (VAS); Knee scores (HSS); ROM
Mu et al. (2022)	Before and after self-comparison	Osteoarthritis of the knee	13	-	65.1 ± 8.8	-	12 months	Knee scores (KSS)
Xu et al. (2022)	Retrospective case-control	Osteoarthritis of the knee	34	31	65.7 ± 6.7	68.5 ± 9.6	6 weeks	Knee scores (KSS); ROM
Ma et al. (2024)	Retrospective case-control	Osteoarthritis of the knee	22	26	68.68 ± 7.92	66.15 ± 6.56	6 months	Pain (VAS); Knee scores (AKS); FJS scores
Nam et al. (2022)	Retrospective case-control	Knee internal rotation	154	154	70.8 ± 6.1	70.7 ± 6.3	12 months	HKA angle

MA-TKA, is MAKO, Robot-Assisted TKA, CM-TKA, is Conventional TKA, VAS is Visual Analog Score; AKS, is American Knee Society Score; KSS, is American Knee Society Score; HSS, is Hospital for Specialty Surgery Knee Score; WOMAC, is Osteoarthritis Index Score; ROM, is Joint Mobility, and FJS, score is forgotten joint score.

**TABLE 2:** Methodological quality assessment of included studies using the JBI Checklist for Quasi-Experimental Studies.

Entry	Ma et al. (2024)	Liu et al. (2024)	Zhang et al. (2023)	Liu et al. (2023)	Ru and He (2023)	Zhang et al. (2022)	Mu et al. (2022)	Xu et al. (2022)	Nam et al. (2022)
1. Was the causal research question clearly defined?	Y	Y	Y	Y	Y	Y	Y	Y	Y
2. Was a control group established?	Y	Y	Y	Y	NA	Y	NA	Y	Y
3. Was the comparability of baseline characteristics between groups described?	Y	Y	Y	Y	NA	Y	NA	Y	Y
4. Were potential confounding factors identified and described?	Y	Y	Y	Y	N	N	N	Y	Y
5. Were the interventions for both groups described in detail?	Y	Y	Y	Y	Y	Y	Y	Y	Y
6. Was blinding applied to participants, personnel, or outcome assessors?	N	N	N	N	N	N	N	N	U
7. Were follow-up data reported and any attrition explained?	Y	Y	Y	Y	Y	Y	Y	Y	Y
8. Were outcome measures reliable?	Y	Y	Y	Y	Y	Y	Y	Y	Y
9. Were appropriate statistical methods used?	Y	Y	Y	Y	Y	Y	Y	Y	Y

JBI, joanna briggs institute; Y, yes; N, no; U, unclear; NA, not applicable.

### Meta-analysis results

**FIGURE 2:**
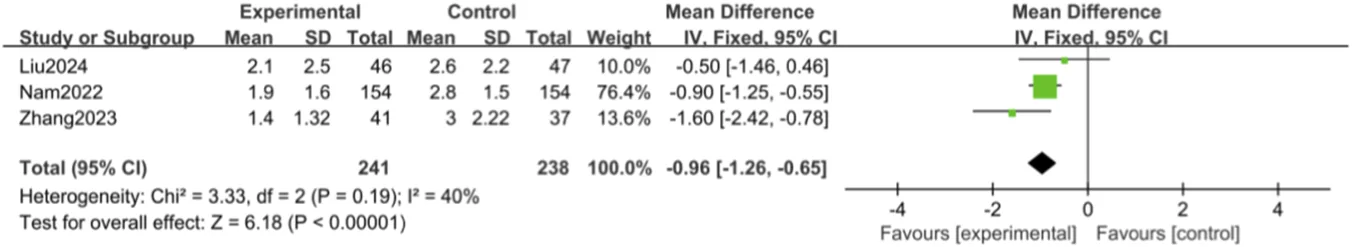
Meta-analyzed forest plot of HKA angle.

HKA angle: Three studies (Liu et al., 2024; Zhang et al., 2023; Nam et al., 2022) reported on the hip-knee-ankle (HKA) angle, with low heterogeneity among studies (P = 0.19, I^2^ = 40%); therefore, a fixed-effects model was employed. The results indicated a statistically significant difference in the HKA angle between the two groups (MD = −0.96, 95% CI –1.26 to −0.65, P < 0.0001), as shown in Figure 2.

**FIGURE 3:**
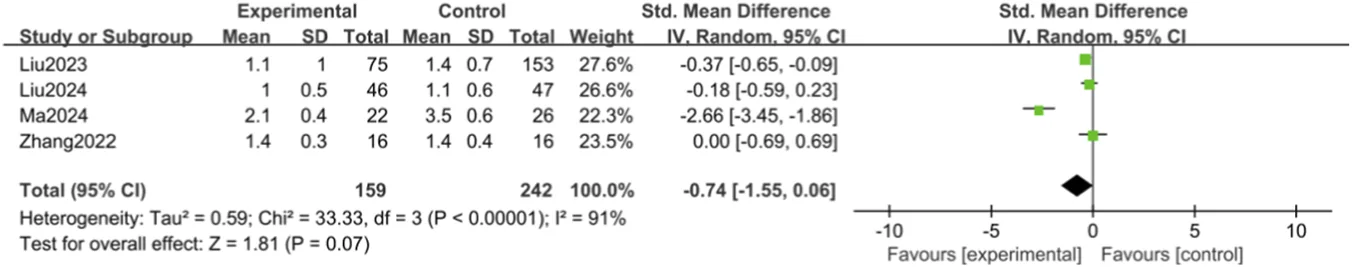
Forest plot for Meta-analysis of pain.

Pain: Four studies (Ma et al., 2024; Liu et al., 2024; Liu et al., 2023; Zhang et al.) reported on pain, with substantial heterogeneity among studies (P < 0.00001, I^2^ = 91%); consequently, a random-effects model was adopted. The results suggested that pain in the experimental group was lower than that in the control group, but the difference was not statistically significant (SMD = −0.74, 95% CI –1.55 to 0.06, P = 0.07), as shown in Figure 3. Another self-controlled study reported pain outcomes (Thomas et al., 2022), showing a significant reduction in pain scores following the intervention compared with baseline levels (SMD = −9.35, 95% CI: −10.48 to −8.21). This finding suggests that the intervention may have a potential role in alleviating pain symptoms.

**FIGURE 4:**
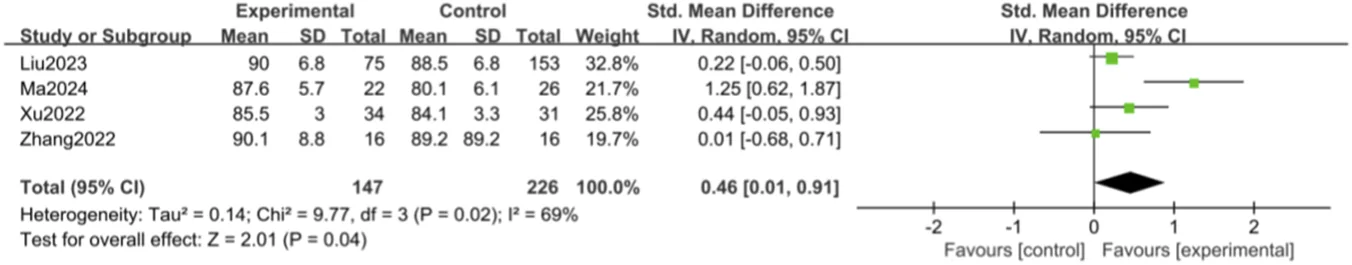
Forest plot of Meta-analysis of knee scores.

**FIGURE 5:**
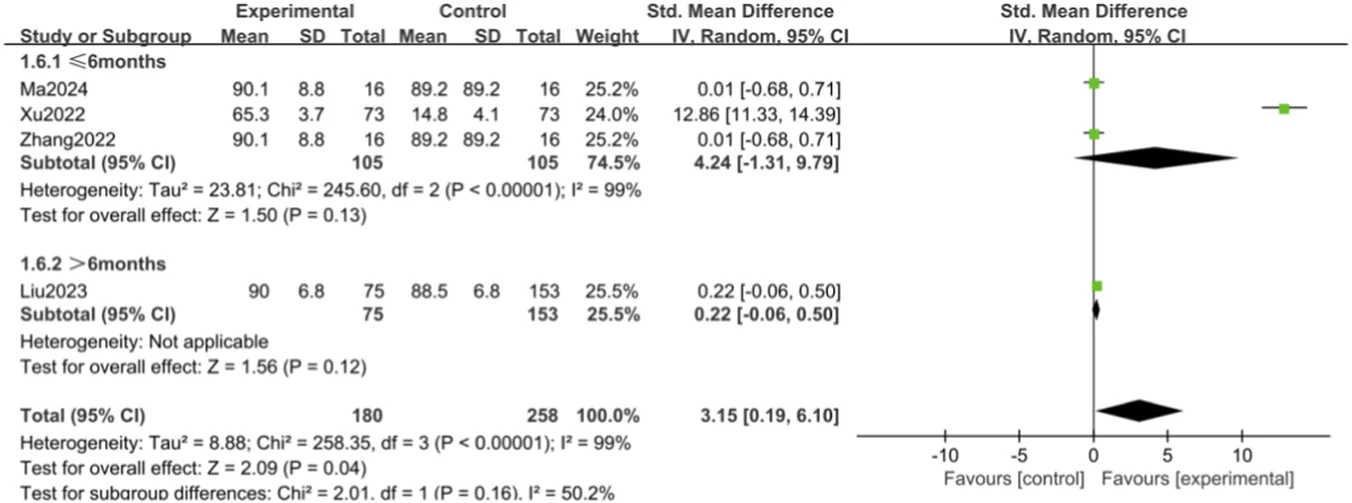
Forest plot of subgroup analysis of knee scores follow-up time.

Knee scores: Four studies (Ma et al., 2024; Liu et al., 2023; Zhang et al.; Xu et al., 2022) reported knee scores. Substantial heterogeneity was observed across studies (P < 0.00001, I^2^ = 98%), and a random-effects model was applied. The results indicated that patients in the experimental group had significantly higher knee scores than those in the control group (SMD = 0.46, 95% CI: 0.01 to 0.91, P = 0.04) (Figure 4). In addition, a subgroup analysis was performed based on follow-up duration, which was considered a potential source of the high heterogeneity. The results showed that for studies with follow-up ≤6 months, knee scores in the experimental group were higher than those in the control group, but the difference was not statistically significant (SMD = 4.24, 95% CI: −1.31 to 9.79, P = 0.13); for studies with follow-up >6 months, the between-group difference in knee scores reached statistical significance (SMD = 3.15, 95% CI: 0.19 to 6.10, P = 0.04). However, the test for subgroup differences did not reach statistical significance, suggesting that current evidence is insufficient to confirm that follow-up duration accounts for the heterogeneity in knee score outcomes (Figure 5). Furthermore, two self-controlled studies (Ru and He, 2023; Mu et al., 2022) evaluated changes in knee function scores, both demonstrating significant improvements from baseline following the intervention. One study reported an SMD of 13.00 (95% CI: 9.09–16.92) for knee score improvement, while the other showed an SMD of 12.86 (95% CI: 11.33–14.39). These findings collectively suggest that the intervention may contribute to improved knee function.

**FIGURE 6:**
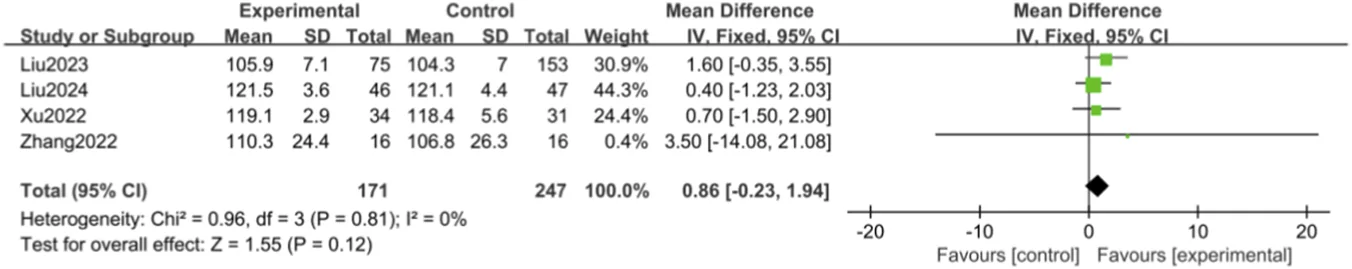
Forest plot for Meta-analysis of range of motion.

ROM: Four studies (Liu et al., 2024; Liu et al., 2023; Zhang et al.; Xu et al., 2022) reported on ROM, with no heterogeneity among studies (P = 0.81, I^2^ = 0%); therefore, a fixed-effects model was applied. The results indicated no statistically significant difference in ROM between the two groups (MD = 0.86, 95% CI: −0.23 to 1.94, P = 0.12) (Figure 6).

**FIGURE 7:**
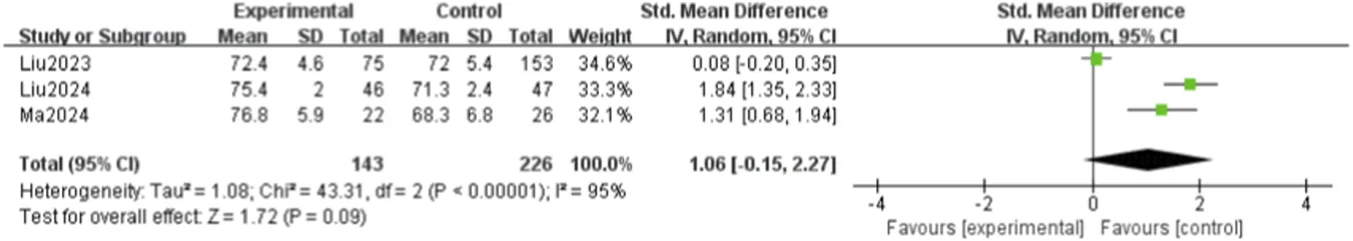
Forest plot of Meta-analysis of FJS scores.

FJS score: Three studies (Ma et al., 2024; Liu et al., 2024; Liu et al., 2023) reported on this outcome, with substantial heterogeneity among studies (P < 0.00001, I^2^ = 95%); thus, a random-effects model was adopted. The results showed no statistically significant difference in FJS scores between the two groups (SMD = 1.06, 95% CI: −0.15 to 2.27, P = 0.09) (Figure 7).

**FIGURE 8:**
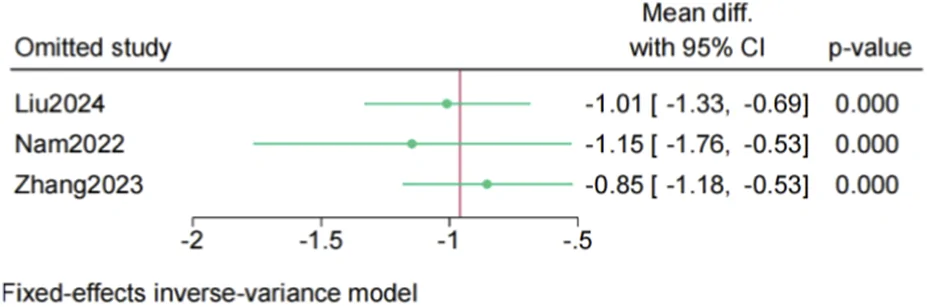
Sensitivity analysis of the HKA angle.

**FIGURE 9:**
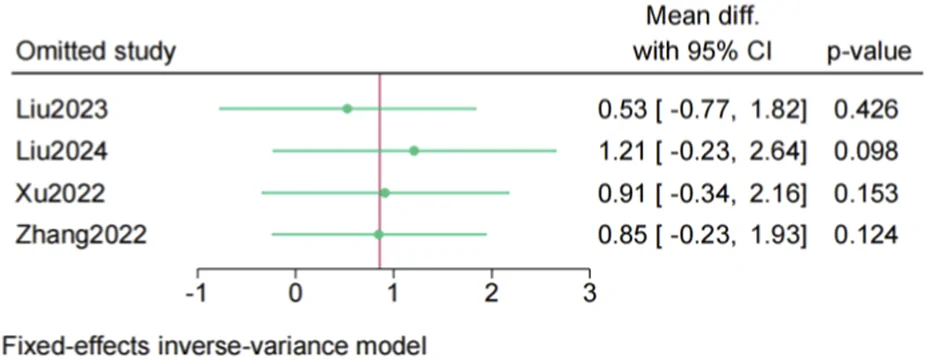
Sensitivity analysis of ROM.

**FIGURE 10:**
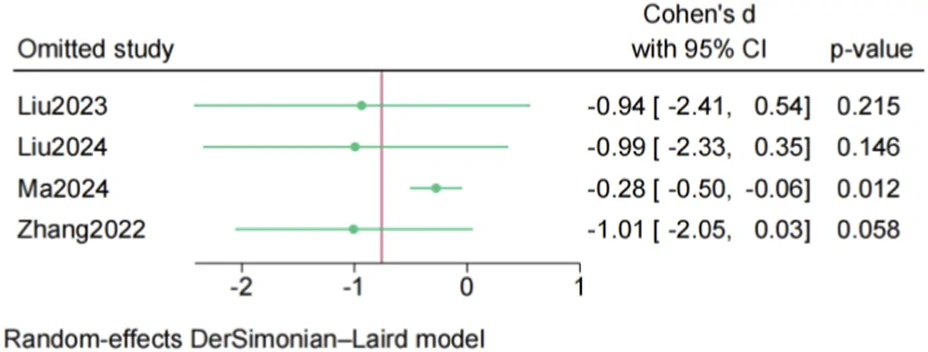
Sensitivity analysis of pain.

**FIGURE 11:**
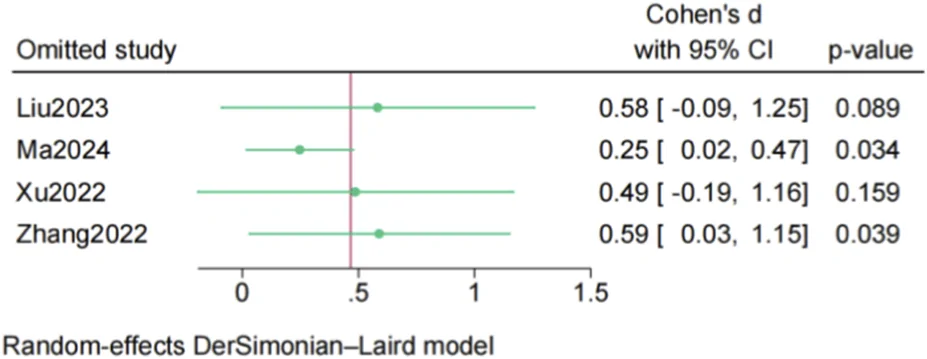
Sensitivity analysis of the knee score.

**FIGURE 12:**
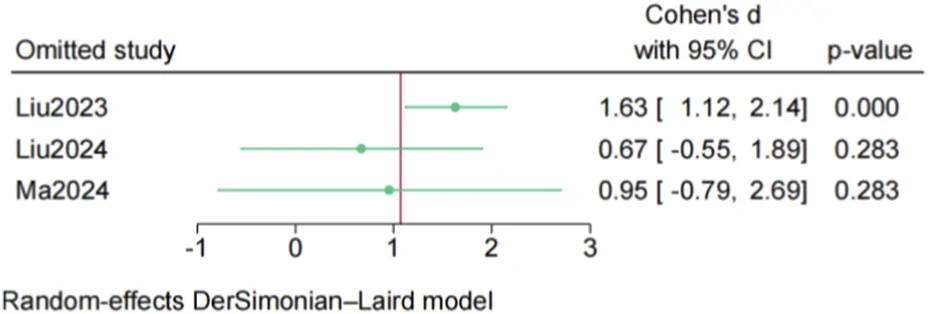
Sensitivity analysis of the FJS.

Sensitivity analysis: A leave-one-out sensitivity analysis was performed. The results demonstrated that for the HKA angle and ROM, sequential exclusion of any single study did not materially alter the pooled effect sizes or statistical conclusions, indicating good robustness of the meta-analysis findings (Figures 8, 9). In contrast, for pain, knee function scores, and FJS outcomes, exclusion of certain studies resulted in significant changes in the results, suggesting a lack of robustness for these endpoints (Figures 10–12).

### GRADE assessment

The quality of evidence for the primary clinical outcomes was evaluated according to the GRADE (Grading of Recommendations Assessment, Development, and Evaluation) criteria. Given that the included evidence was predominantly derived from non-randomized comparative studies, the initial certainty of evidence for all outcome measures was considered to be low. After comprehensive consideration of study design limitations, heterogeneity, imprecision, and variations in outcome measurement, the certainty of evidence was rated as low for the HKA angle and ROM outcomes. For pain, knee score, and FJS, the certainty of evidence was downgraded to very low due to substantial heterogeneity, imprecision of results, or differences in the assessment tools used across studies. The detailed assessment results are presented in Table 3.

**TABLE 3:** GRADE assessment results.

Outcome measure	Number of studies (Patients)	Effect estimate	Risk of bias	Inconsistency	Indirectness	Imprecision	Publication bias	Certainty of evidence
HKA angle	3 studies (580 patients)	MD = −0.96,95%CI -1.26∼-0.65	Downgraded 1 level (non-randomized studies)	Not downgraded (I^2^ = 40%)	Not downgraded	Not downgraded (narrow CI, consistent direction of effect)	Not downgraded (insufficient studies to assess reliably)	⊕⊕○○
Pain (VAS)	4 studies (472 patients)	SMD = −0.74,95%CI -1.55∼0.06	Downgraded 1 level (non-randomized studies)	Downgraded 1 level (I^2^ = 91%, substantial heterogeneity)	Not downgraded	Downgraded 1 level (CI crosses null, imprecise estimate)	Not downgraded	⊕○○○
Knee Function Scores	4 studies (388 patients)	SMD = 0.46,95%CI 0.01∼0.91	Downgraded 1 level (non-randomized studies)	Downgraded 1 level (I^2^ = 98%, substantial heterogeneity)	Downgraded 1 level (different instruments: AKS/KSS/HSS/WOMAC; construct validity concerns)	Downgraded 1 level (CI approaches null)	Not downgraded	⊕○○○
ROM	4 studies (374 patients)	MD = 0.86,95%CI -0.23∼1.94	Downgraded 1 level (non-randomized studies)	Not downgraded (I^2^ = 0%)	Not downgraded	Downgraded 1 level (CI includes null; limited sample size)	Not downgraded	⊕⊕○○
FJS Score	3 studies (326 patients)	SMD = 1.06,95%CI -0.15∼2.27	Downgraded 1 level (non-randomized studies)	Downgraded 1 level (I^2^ = 95%, substantial heterogeneity)	Not downgraded	Downgraded 1 level (wide CI crossing null)	Not downgraded	⊕○○○

## Discussion

The results of this study showed that patients with MAKO robot-assisted TKA were better than patients with conventional TKA in terms of HKA angle and knee scores, and the differences were all statistically significant (P < 0.05); however, the differences between the two groups in terms of ROM, pain, and FJS scores were not statistically significant (P > 0.05).

The results of this study showed that MAKO robot-assisted TKA patients were superior to conventional TKA patients in terms of HKA angle. These findings are consistent with those reported by Lu et al. (Lu et al., 2024). This concordance may be attributed to the MAKO robot’s advanced three-dimensional preoperative planning system, which enables individualized surgical planning based on the patient’s knee CT data, thereby facilitating precise preoperative and intraoperative adjustments. Intraoperatively, the real-time navigation capability of the MAKO robot further ensures the accuracy of component positioning, minimizing errors associated with manual techniques. Consequently, this optimizes the lower-limb mechanical axis, contributing to a more favorable HKA angle. The results of a study by Ng et al. (2024) found that patients who underwent MAKO robot-assisted TKA experienced lower postoperative pain compared to patients in the conventional group. The reason for this may be that MAKO robot-assisted TKA surgery utilizes minimally invasive techniques, which reduces surgical trauma and bleeding. This minimally invasive procedure helps to reduce postoperative pain and speeds up the recovery process. However, this finding was not confirmed in the pooled analysis of the present study.

The data of this study showed that MAKO robot-assisted TKA patients were superior to conventional TKA patients in terms of knee scores. This is consistent with the findings of Baek et al. (2023). Due to the greater precision and minimally invasiveness of MAKO robot-assisted TKA surgery, patients were able to regain knee function more quickly after surgery, which in turn led to a significant improvement in knee scores. On the other hand MAKO robot-assisted TKA effectively relieves postoperative pain and improves patient satisfaction, which is one of the important factors for the improvement of knee scores (Ping et al., 2024). A subgroup analysis based on follow-up duration for knee function scores revealed that patients undergoing MAKO robot-assisted TKA achieved higher knee scores than those undergoing conventional TKA in both the >6 months and ≤6 months follow-up groups. However, the difference between the two groups did not reach statistical significance in the ≤6 months subgroup. These findings suggest that MAKO robot-assisted TKA may offer advantages over conventional TKA in both the short-term and long-term follow-up periods, although further studies with larger sample sizes are warranted to confirm these observations. MAKO robot-assisted TKA has high precision during the surgical procedure, which ensures accurate implantation of the prosthesis and optimizes the knee joint line of force, and the minimally invasive procedure reduces trauma and recovery time, which helps to accelerate the recovery process. Although the initial recovery of traditional TKA patients may be slower, their knee function also gradually improves during the long-term rehabilitation process (Han et al., 2024), leading to a gradual convergence of the rehabilitation process between the two groups. Studies comparing the long-term effects of MAKO robot-assisted TKA with conventional TKA are still relatively limited. Therefore, more long-term, large-sample, multicenter studies are needed in the future to further validate the difference in long-term outcomes between the two and to explore factors that may affect long-term outcomes.

The results of this study suggest that MAKO robot-assisted TKA cannot yet be considered to improve ROM, pain, and FJS scores in patients with knee disorders compared to conventional TKA. These results contradict the findings of Liu et al. (2024). Ningning Liu found that patients with MAKO robot-assisted TKA had significantly higher ROM at 3 months postoperatively and FJS scores at 6 months postoperatively compared with conventional TKA. However, the surgical outcome may also be affected by a variety of factors, such as patient’s age, severity of disease, and postoperative rehabilitation, and more large-sample, high-quality studies are needed to validate this.

The findings of several previous studies are consistent with the present study in multiple aspects, namely, that robotic-assisted TKA demonstrates more consistent advantages in radiographic alignment parameters, while the differences in functional scores, pain improvement, and long-term range of motion are less significant or inconsistent. One meta-analysis indicated that robotic-assisted TKA showed certain advantages in mechanical alignment and WOMAC scores, but did not demonstrate consistent improvement in ROM, suggesting the need for more long-term data to assess long-term prognosis (Daoub et al., 2024). Another recent systematic review also noted that robotic-assisted osteotomy techniques may be superior to conventional techniques in short-term clinical and radiographic outcomes, but their effects on long-term function, pain relief, and cost-effectiveness remain unclear (Wada et al., 2025). Furthermore, a review summarizing evidence from existing RCTs and cohort studies concluded that while robotic-assisted TKA can improve the precision of prosthesis implantation, this does not necessarily translate into consistent clinical advantages, and heterogeneity in results may arise from differences in follow-up duration and outcome measure selection (Fu et al., 2024).

At present, there remains insufficient evidence regarding the long-term clinical benefits and cost-effectiveness of MAKO robot-assisted TKA. Although existing randomized controlled trials have demonstrated that robot-assisted technology can improve prosthetic positioning and restore limb alignment with greater accuracy, it has not yet shown a clear advantage over conventional TKA in terms of long-term functional scores or prosthetic survival. This suggests that improvements in radiological parameters may not necessarily translate into sustained long-term patient benefits (KIM et al., 2020). A recent systematic review focusing specifically on the MAKO system similarly noted that robot-assisted TKA may improve certain mid-term functional outcomes, but that its long-term efficacy still requires validation through more high-quality studies (Sodhi et al., 2025). Furthermore, the economic value of robot-assisted TKA remains controversial. While some studies suggest that it may be cost-effective in high-volume centers, these findings are highly dependent on factors such as the healthcare system, device costs, and surgical volume (Hua and Salcedo, 2022). Therefore, future research should not only focus on short-term radiological and functional indicators, but also further evaluate long-term prosthetic survival, revision rates, patient-reported satisfaction, and health economic outcomes.

In summary, MAKO robot-assisted TKA demonstrates superior efficacy in improving the HKA angle and knee function scores in patients with knee joint diseases compared with conventional TKA. However, current evidence does not support the conclusion that MAKO robot-assisted TKA offers additional advantages over conventional TKA in terms of improving postoperative ROM, pain relief, or FJS scores.

However, this meta-analysis has several limitations. Although the quality of the included studies was relatively high, all of them were non-randomized designs, which may introduce selection bias and confounding effects. Therefore, the conclusions should be interpreted with caution, and high-quality RCTs are still needed for further validation. The included studies employed a wide variety of knee scoring systems for postoperative evaluation following TKA, which precluded subgroup analysis based on the assessment tools. The relatively small number of included studies and limited sample sizes may increase the risk of bias and prevented us from performing robust subgroup analyses to identify the sources of heterogeneity. Furthermore, sensitivity analyses for some outcome measures revealed changes in statistical significance, suggesting that certain findings lack robustness. In this study, we further adopted the Grading of Recommendations Assessment, Development and Evaluation (GRADE) framework to assess the certainty of evidence. Although some meta-analytic results demonstrated statistically significant advantages of MAKO robot-assisted TKA in terms of the HKA angle and knee function scores, the overall certainty of evidence was low, largely due to the predominant reliance on non-randomized comparative studies and the substantial heterogeneity observed in certain outcomes. These findings indicate that the current evidence supporting the clinical benefits of MAKO robot-assisted TKA should be interpreted with prudence. Consequently, high-quality, large-sample randomized controlled trials are warranted to corroborate the conclusions of the present study. Additionally, by restricting inclusion to Chinese and English publications, potentially relevant studies in other languages may have been omitted, introducing possible publication bias. To enhance the scientific rigor and generalizability of future research in this field, we recommend that subsequent studies adopt multicenter, large-sample, high-quality RCT designs, standardize outcome assessment tools, and strive to incorporate literature in multiple languages and grey literature from trial registries, thereby improving the comprehensiveness and representativeness of the evidence.

## Conclusion

Compared with conventional TKA, MAKO robot-assisted TKA demonstrates certain advantages in terms of the HKA angle and knee function scores. However, given that the current evidence is predominantly derived from observational studies, with notable heterogeneity and potential confounding factors, these findings should be interpreted with caution. At present, there is insufficient evidence to support a definitive superiority of MAKO robot-assisted TKA over conventional TKA in terms of pain relief, ROM, or the FJS. Future high-quality, multicenter, prospective studies with long-term follow-up are warranted to further validate its clinical benefits and long-term efficacy.

## Data Availability

The original contributions presented in the study are included in the article/Supplementary Material, further inquiries can be directed to the corresponding author.
