# Supplementary material for: Efficacy of MAKO robot-assisted total knee arthroplasty compared with conventional total knee arthroplasty in patients with knee joint diseases: a systematic review and meta-analysis
Source: Front Bioeng Biotechnol. 2026 Aug 18;14:1892225. doi: 10.3389/fbioe.2026.1892225 (PMC13529720; doi:10.3389/fbioe.2026.1892225)
Supplement: Supplementary file 1 [file Table1.docx]

Supplementary Table 1 Detailed search strategy

| Database | Search Strategy (Topic Section) | Results |
| --- | --- | --- |
| PubMed | ("Arthroplasty, Replacement, Knee"[Mesh] OR "total knee arthroplasty"[Title/Abstract] OR "total knee replacement"[Title/Abstract]) AND ("MAKO robotic-assisted"[Title/Abstract] OR "robotic-assisted"[Title/Abstract]) | 1108 |
| Embase | ('arthroplasty replacement knee'/exp OR 'total knee arthroplasty':ti,ab,kw OR 'total knee replacement':ti,ab,kw) AND ('mako robotic-assisted':ti,ab,kw OR 'robotic-assisted':ti,ab,kw) | 1055 |
| Scopus | TITLE-ABS-KEY("Arthroplasty, Replacement, Knee" OR "total knee arthroplasty" OR "total knee replacement") AND TITLE-ABS-KEY("MAKO robotic-assisted" OR "robotic-assisted" OR MAKO) | 1265 |
| Web of Science | TS=("Arthroplasty, Replacement, Knee" OR "total knee arthroplasty" OR "total knee replacement") AND TS=("MAKO robotic-assisted" OR "robotic-assisted" OR MAKO) | 955 |
| Cochrane Library | ("Arthroplasty, Replacement, Knee" OR "total knee arthroplasty" OR "total knee replacement") AND ("MAKO robotic-assisted" OR "robotic-assisted") | 136 |
| CNKI (Subject) | SU = ('Total Knee Arthroplasty') AND (SU = ('MAKO Robot-Assisted') OR SU = ('MAKO-assisted')) | 80 |
| Wanfang (Professional Search) | Subject: (Total Knee Arthroplasty) AND Subject: (MAKO Robot-Assisted OR MAKO-assisted) | 61 |
| VIP (Search Formula Retrieval) | M = (Total Knee Arthroplasty) AND (M = (MAKO Robot-Assisted OR MAKO-assisted)) | 120 |
